# Phage-Displayed Peptides for Targeting Tyrosine Kinase Membrane Receptors in Cancer Therapy

**DOI:** 10.3390/v13040649

**Published:** 2021-04-09

**Authors:** Annamaria Aloisio, Nancy Nisticò, Selena Mimmi, Domenico Maisano, Eleonora Vecchio, Giuseppe Fiume, Enrico Iaccino, Ileana Quinto

**Affiliations:** Department of Experimental and Clinical Medicine, University Magna Graecia of Catanzaro, 88100 Catanzaro, Italy; nancynistico@unicz.it (N.N.); mimmi@unicz.it (S.M.); maisano@unicz.it (D.M.); eleonoravecchio@unicz.it (E.V.); fiume@unicz.it (G.F.); iaccino@unicz.it (E.I.)

**Keywords:** phage display, peptide ligands, tyrosine kinase receptors, tumor-targeting

## Abstract

Receptor tyrosine kinases (RTKs) regulate critical physiological processes, such as cell growth, survival, motility, and metabolism. Abnormal activation of RTKs and relative downstream signaling is implicated in cancer pathogenesis. Phage display allows the rapid selection of peptide ligands of membrane receptors. These peptides can target in vitro and in vivo tumor cells and represent a novel therapeutic approach for cancer therapy. Further, they are more convenient compared to antibodies, being less expensive and non-immunogenic. In this review, we describe the state-of-the-art of phage display for development of peptide ligands of tyrosine kinase membrane receptors and discuss their potential applications for tumor-targeted therapy.

## 1. The Evolution of Phage Display

**Figure 1:**
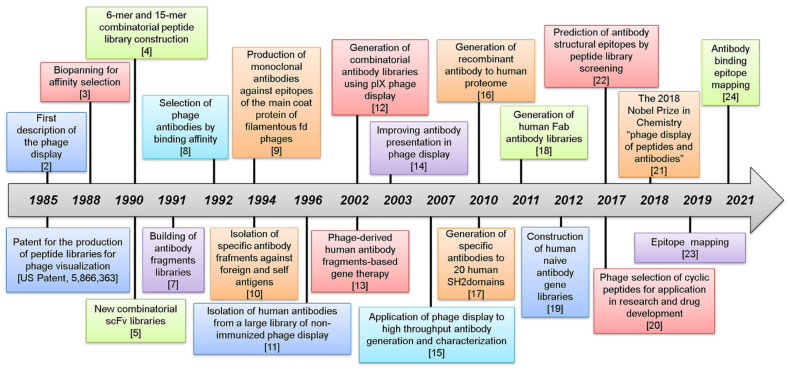
Milestones of phage display technique from discovery to date.

Phage display represents a useful technique for studying protein–protein interactions that regulate the biological processes [1]. Bacteriophages, which are viruses infecting bacteria, can express recombinant peptides on their surface coat following the cloning of random short DNA sequences within their genome. The phage display technique was first described in 1985 by George P. Smith, who demonstrated the expression of a foreign insert on a filamentous phage surface following its cloning in frame with the minor coat protein pIII [2]. In the same year, George Pieczenik patented the production of random peptide libraries for phage display (US Patent, 5,866,363). In 1988, the selection of phage ligands of target proteins was improved by using a process called “biopanning”, which significantly reduced antibody requirements compared to the original procedure published in 1985 [3]. In the 1990s, combinatorial phage libraries containing 40 million 6-mer peptides [4] or 20 million 15-mer peptides [5] were built. As predicted by Smith, the use of these libraries allowed an effective investigation of the specific affinity binding to antibody epitopes, receptors, or other proteins using simple recombinant DNA methods. What Smith did not imagine was the wide number of applications of his invention in various biomedical fields. The phage display technology was further developed and improved by the following research teams: G. Winter and J. McCafferty of the Medical Research Council, Laboratory of Molecular Biology; R. Lerner and C. Barbas of the Scripps Research Institute; F. Breitling and S. Dübel of the German Cancer Research Center [6,7,8,9,10,11]. All these researchers pursued the creation of phage-displayed combinatorial antibodies libraries, which were further improved by several other laboratories in the following years [12,13,14,15,16,17,18,19,20]. As recognition of the phage display contribution to scientific advances in chemistry and pharmaceutics, George P. Smith and Sir Gregory P. Winter received the 2018 Nobel Prize in Chemistry “for phage visualization of peptides and antibodies” [21]. More recently, the phage display has been useful for the mapping of antibody binding epitope and the screening of combinatorial peptide libraries in drugs discovery [22,23,24]. A timeline of phage display development is shown in Figure 1. 

Nowadays, the phage display allows the production of vast libraries of peptides displayed on the surface of phage particles, which can differ for quantities and length of peptides, allowing the selection of binding partners for several applications in medicine, biotechnology, and pharmacology [25]. Among the enormous diversity of peptide or protein variants shown in a phage library (10^9^–10^10^ different phages), those with high affinity and specificity for the target are isolated through affinity selection loops [26]. In this way, large amounts of peptide ligands can be quickly identified and amplified by an in vitro selection process called “panning”. Phage clones selected for high affinity binding to the bait can be easily sequenced at the genomic level in order to determine the amino acid sequence of the peptide insert required for the binding [27]. The high flexibility of phages to display proteins with various sizes and properties without prior knowledge of their structure and sequence has led to new applications. Hence, a modern era in drug discovery has emerged concurrently with the development of phage display technology.

## 2. Phages Used for Peptide Display

The bacteriophage genome can be genetically modified in order to express specific amino acid sequences within the coating proteins of the surface [28]. The first experimental approach of George Smith was based on the use of the filamentous phage M13 [2]. Later, phages such as T4 [29], T7 [30], and lambda [31] were also used. However, the most used phage display system is based on lysogenic filamentous phages, such as M13 [32,33]. The term lysogenic refers to the ability of bacteriophages to remain silently integrated within the bacterial genome (prophage state) upon infection. Lysogenic phages can be expanded by culturing the host bacteria in order to create peptide libraries of up to 10^10^ different fusion variants for the following purposes: (i) analysis of protein-binding interactions; (ii) identification of binding site for receptor and antibody; (iii) identification of epitopes for monoclonal antibodies; (iv) identification of the enzyme-substrate interaction. M13 phages belong to the F-positive phage family and infect *Escherichia coli* as host. During infection, the phage M13 absorbs on the bacterial pilus through its pIII capsid protein. The single-stranded DNA (ssDNA, also named “+ strand”) penetrates the cell and is converted into a circular double-stranded molecule, named “replicative form” (RF). When about 100–200 copies of the phage have been synthesized, pV binds to the + single strands and prevents them from replicating. The filaments can thus interact with the cell membrane through pVII and pIX, and once the contact is made, pV is replaced by pVIII during deportation, and pIII is added at one proximal end of the phage. The assembled phage is extruded from the host cells without lysing it. The first bacterial infection produces about 1000 phages, and this number decreases to 100–200 particles in the next generations. As a disadvantage, the M13 phage exhibits a physical limitation because it requires periplasmic transport of the peptide/protein library during amplification and has low efficiency for the visualization of cytoplasmic proteins [34]. In the 1990s, the phage display technique also used the bacteriophages T7 and T4 with appropriate modifications [35,36]. Advantages of using these bacteriophages in place of M13 phage include the ability to display large proteins due to the ability of these phages to carry 40 up to 160 kb DNA insert in their genome [34], and the ability to be released from the host cell following lysis. Unlike M13, the phage T7 has a lytic cycle and can bind the *E. coli* host even if the capsid protein is involved in the binding to the bait. This allows the rapid phage recovery and amplification without carrying out the elution step [37,38]. Further, the inserted peptide is displayed at the C terminal of the capsid protein without requiring the removal of the stop codon. Bacteriophage T4 can visualize foreign polypeptides using fusion with non-essential proteins of the capsid (SOC and HOC). Instead, the lambda phage has a life cycle that allows it to reside as prophage into the host genome or to enter the lytic phase, during which it kills and lyses the bacterium to produce offspring [39]. The main characteristics of the various phages are summarized in Table 1. 

## 3. Biopanning

The screening of the phage displayed peptide library consists of sequential steps of phages selection for affinity binding to the bait, so called “biopanning”. To build a random peptide library, it is first necessary to clone the DNA sequences of random peptides in frame with the sequence of the bacteriophage coat protein and to amplify the resulting phage library in the host bacteria [40]. The recombinant phages are incubated with the bait molecule to allow their binding through the displayed peptides. Once the incubation time elapses, the unbound and non-specific phages are removed using sequential washings with increased stringency conditions. The phages linked to the bait molecule are then separated by competitive elution with natural ligands or by washing with acidic buffer. To optimize the screening strategy, other parameters must be considered in the selection steps: (i) washing buffer stringency by increasing saline or detergent concentration to select peptides with higher affinity; (ii) determining the yield of phages with a high affinity binding that are still able to survive [32]. Incubation time of the phages with the target, number of washing steps, elution conditions, and target concentration/density are all to be considered for an accurate selection of phage binders [41,42].

**Figure 2:**
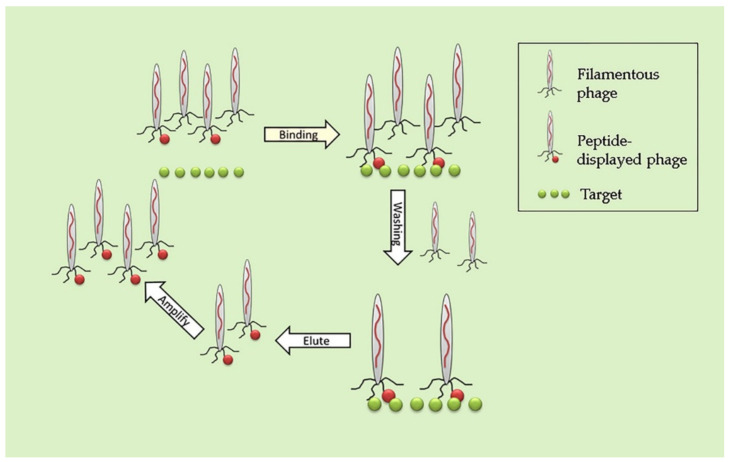
Screening of a phage-displayed random peptide library using a protein as bait.

The eluted phages are then amplified by bacterial infection and again incubated with the bait to enrich the number of phage binders by several steps of affinity binding [40]. It has to be considered that the phages that display peptide sequences that disable the correct folding of the phage membrane are lost during the rounds of amplification by biopanning because of the lack of infectivity (Figure 2). 

The phage ligands are generally selected by 3–5 cycles of affinity-driven biopanning, followed by sequencing of their DNA insert to determine the primary structure of the displayed peptides [43]. 

## 4. Phage Display for Selection of Peptide Ligands of Membrane Receptors

The screening of a phage-displayed peptide library involves the risk of selecting peptides that are unable to maintain their binding specificity when isolated outside of the phage context or when tested for the binding to the natural bait in the physiological context [44]. For example, the selection of peptide ligands of membrane receptors as baits poses the question of how to preserve the conformation of the receptor detached from the cellular membrane. In fact, the conformation of a membrane receptor as purified recombinant protein may differ from the native conformation exposed on the cell surface. To this end, the screening of a phage-displayed peptide library must be adjusted to the physiological in vitro and in vivo conditions for selecting peptide ligands with high affinity and specificity for the target receptor.

The in vitro whole-cell screening is very useful for identifying numerous peptides that bind specifically to membrane receptors of cell lines and primary cells in culture, or fixed cells. The choice between live or fixed cells depends on the final application of the selected ligand. By this experimental procedure, the maintenance of the biological functions, expression level, and three-dimensional structure of the membrane receptors as bait are preserved. Thus, the whole-cell screening by phage display allows the selection of peptides that are able to bind the native membrane receptors in physiological conditions and to compete with natural ligands [45,46]. 

The screening of peptide ligands can be also performed by intravenous injection of phage-displayed random peptide libraries in animals, allowing the in vivo binding of phages to the target cells [47,48]. Following in vivo incubation, the animals are perfused to remove unbound phages and then sacrificed. Chosen organs are harvested and homogenized in order to isolate the bound phages, which are then amplified and enriched by additional rounds of in vivo selection. By this approach, peptide ligands of different cancer types as well as tissue-specific or tumor vasculature-homing peptides have been identified [49,50,51]. For instance, vasculature targeting peptides containing RGD (arginine/glycine/aspartic acid) motif specifically bind to integrins or cell surface molecules or receptors overexpressed on tumor blood vessels [49]. The cyclic peptide CNGRC containing the NGR (asparagine/glycine/arginine) motif selectively targets angiogenic endothelial cells [52]. Another peptide, TLTYTWS—binding to collagen IV and processed by proteolytic activity of MMP-2—has been identified by phage display and showed the inhibition of tumor-homing capabilities in a lung carcinoma mouse model, with consequent antiangiogenic effects [53]. Further investigations on intra-species differences of peptide binding are required to improve the translational application of peptide ligands from the animal models to humans [46].

## 5. Phage Display for Analysis of Protein–Protein Interaction Domains

Increasing knowledge on the molecular basis of protein–protein interaction has facilitated the discovery, design, and development of novel drugs for targeted therapy in human diseases. In addition to the selection of membrane receptors peptide ligands, the phage display represents an efficient method for the analysis of other protein–protein interactions that are mediated by highly conserved modular protein domains. In particular, the SrC homology (SH) 2 and 3 domains are relevant as they bind tyrosine phosphorylated target proteins acting in the downstream signaling of tyrosine kinase receptors [1]. Particularly relevant has been the use of phage display to study the SH3 domain-mediated protein–protein interaction. The SH3 domain contains highly conserved protein modules of 50–70 amino acids, which interact with adaptor and tyrosine kinase proteins involved in signaling pathways regulating the proliferation and cytoskeleton organization. The SH3 domain usually binds proline residues in a specific conformation termed “proline-rich domains” (PRD) and folds into a globular structure. Karkkainen et al. generated a library displaying all human SH3 domains in M13 bacteriophage coat to identify potential protein partners and identified the interaction with several proteins functionally unrelated [54]. Other scientists focused on the interaction of SH3 domains with intracellular regions of Fas ligand by similar screening and identified numerous additional SH3 binding partners interacting with FasL [55]. Phage display was also used for the screening of peptides able to specifically bind the PSD-95/Discs-large/ZO-1(PDZ) domains, which interact with linear motifs present at the C-terminus of target proteins, allowing the prediction of human PDZ–peptide interactions by bioinformatics [56]. Libraries of peptides and phage display antibodies have been built to define antibody binding sites in isolated immunoglobulins [57], improving the fields of immunotherapy and vaccine development [58].

## 6. Optimization of Peptides for Targeting

The screening of highly diversified peptide libraries by phage display is a new promising approach for drug discovery [59]. In fact, the structure of peptide ligands selected by phage display can be analyzed by bioinformatics in order to identify the chemical interactions with the target molecules, allowing the design of pharmacophores for drugs development [60]. The use of peptides as potential drugs may provide several benefits. Compared to other pharmaceutical molecules, peptides have fewer side effects [61]. They are smaller than antibodies and can be easily synthesized and modified to improve stability, solubility, tissue penetration, and biocompatibility [62,63]. However, the low bioavailability of peptides still represents a weak point that requires further investigation [64]. In perspective, peptides can be powerful tools for tumor monitoring and targeted-therapy when selected for their ability to bind to tumor biomarkers [65]. For example, cytotoxic “pore-forming” peptides (such as those derived from the Bcl-2 family) are promising anti-cancer agents that can be effective against tumor cells at low concentrations while being non-lethal to normal tissue. Among them, most cytotoxic peptides are in pre-clinical stages of testing and have yet to enter clinical trials for cancer [66]. Compared to antibodies, peptides have a higher penetration in solid tumors due to their small size and can target overexpressed membrane receptors, dense extracellular matrixes, and tumor stromal cells [67,68]. An important characteristic of peptides is their affinity binding to the target, i.e., the strength of interaction. This interaction is unique as the peptide is unable to bind to the target through multiple interactions [69]. Further, peptides ligands can be optimized for targeting by combining the phage display-based selection with the computational modeling of peptide structure. The peptide optimization can be achieved by chemical modifications that increase their affinity, avidity, target specificity, and water solubility [70].

Regarding the solubility in water, the peptide length must be considered. Peptides with less than 5 amino acids are more soluble in water compared with longer peptides. Solubility influences the peptide activity and could reduce the ability to maintain a fixed three-dimensional conformation in aqueous solution, thus altering a specific binding to the cognate receptor [71]. To counteract this problem, phage display libraries have been created in order to express the constrained circular structure of the peptides through disulfide bridges between the amino- and carboxy-terminal cysteines. Further, betaine can increase the solubilization of peptides by reducing peptide [72]. Different strategies have been used to improve the pharmacokinetic properties of targeting peptides in vivo. Nanoparticles can be functionalized with peptide ligands for the delivery of miRNAs, siRNAs, or small ncRNAs into target cells or tissues [73]. Small regulatory RNAs, such as microRNAs, can silence their target mRNAs. Nanoparticles can be functionalized with microRNAs in order to interfere with the expression of specific oncogenes promoting tumor growth. Optimization of microRNAs delivery to tumor cells can be achieved by the inclusion of peptide ligands of tumor-receptors within the nanoparticles. In this regard, cationic peptides, protamine, and cell penetrating peptides have emerged as potential components of nanocarriers for targeted delivery of RNAs with low toxicity, as they enhance cell internalization and confer an effective RNA delivery [74].

In fact, the peptide assemblage with nanocarriers increases the likelihood of interacting with specific ligands [75]. The use of peptide ligands with cyclic structure facilitates the correct position of their amino acid sequence with respect to the target epitope. In vitro chemical modifications of these peptides provide greater serum stability with an extended half-life in vivo due to the increased protease-resistance in tissues and serum and to the reduced degradation. Consequently, the peptide dose required for effective action can be reduced, lowering the risk of adverse effects associated with activation of immune response. Peptide ligands can be labeled with fluorescent dyes, such as fluorescein isothiocyanate, for flow cytometry and confocal microscopy in vitro detection of the target molecule expressed on tumor cells, and for ex vivo detection on primary and metastatic tumor cells and tissues. Targeting peptides can also be radiolabeled with different contrast agents (^99^mTc, ^123^I, ^111^In, ^18^F, ^64^Cu, ^68^Ga, ^90^Y and ^177^Lu) for in vivo tumor detection. Moreover, several peptides are under clinical investigation as potential candidates for the diagnosis of different tumors [76]. Examples of these peptides for diagnostic purposes are: HER3P1 peptide labelled with ^68^Ga used as agent for PET imaging; ERBB2-targeted peptide 1-D03 labeled with ^111^In-DOTA used for SPECT imaging of breast carcinoma; and gastrin-releasing peptide, epidermal growth factor, and glucagon-like peptide-1 receptor tested for imaging in early cancer diagnosis. The aforementioned approaches have also been used in our laboratory to monitor the in vitro and in vivo binding of peptide ligands to the immunoglobulin B-cell receptor (IgBCR) of tumor B cells. In particular, a radiolabeled peptide ligand detected in vitro and in vivo A20 murine B-cell lymphoma cells [77]. FITC-conjugated peptide ligands were used to monitor the exosomes secreted in a murine multiple myeloma as biomarkers of tumor growth [78]. Peptide ligands of the IgBCR expressed by chronic lymphocytic leukemia primary cells were also used to monitor tumor populations in disease progression [79].

## 7. Membrane Receptors in Cancer Cells

**Figure 3:**
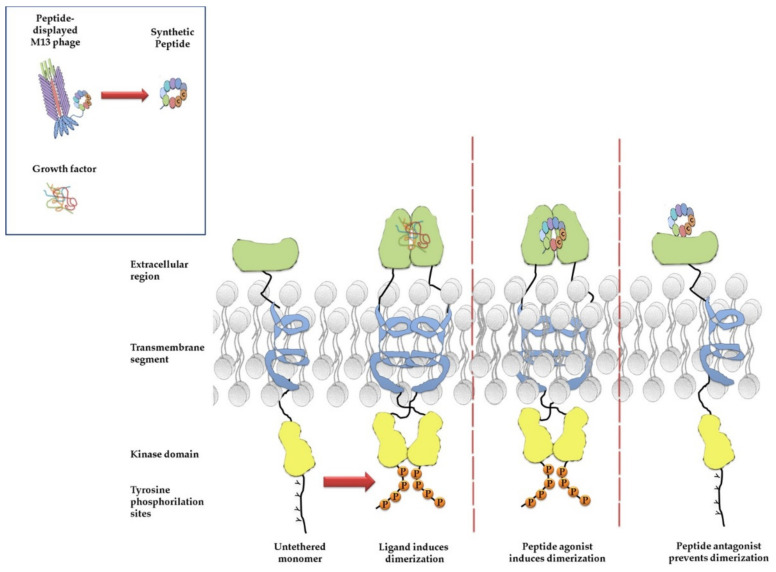
Activation mechanism of a tyrosine kinase receptor. In absence of the ligand, the tyrosine kinase receptor is inactive. The binding of the ligand induces a conformational change of the receptor, causing dimerization and cross-interaction of the dimerization loop within the ectodomain with the ligand. The ligand can be a natural ligand, such as a cytokine or growth factor, or a peptide selected by phage display acting as mimotope of the ligand. The active receptor tyrosine kinase auto-phosphorylates specific tyrosines within the intracellular domain, acting as docking sites for molecules of downstream signaling. A peptide ligand can act as agonist or antagonist of the receptor signaling.

Membrane receptors are central players of biochemical and electrical signaling transduction regulating different cellular processes [80]. Deregulated activity of membrane receptors is often associated with neoplastic transformation [81]. In particular, overexpression of growth factor receptors has a prominent role in tumor growth. In fact, the altered membrane receptor signaling can upregulate the transcription of anti-apoptotic and proliferative genes, causing an unbalance between cell proliferation and cell death, which must be strictly regulated to counteract tumorigenesis [82]. The receptor tyrosine kinases (RTKs) family includes membrane receptors whose tyrosine kinase activity is induced upon the conformational change caused by binding with their specific growth factors. The intracellular signaling of RTKs promotes cell growth, differentiation, survival, migration, and metabolism. To date, 58 different RTKs have been identified and clustered in 20 families. These receptors share a similar molecular architecture that includes: an extracellular region, termed ectodomain, with one or more ligand-binding sites; a hydrophobic transmembrane helix; a juxtamembrane regulatory region; and a cytoplasmic region composed of the tyrosine kinase domain (TKD) and a C-terminal tyrosine-rich tail [83]. As a result of the binding of the growth factor to the receptor extracellular domain, the receptor undergoes conformational changes resulting in the formation of a ligand-bound dimeric complex [84]. Upon the receptor dimerization, the intracellular TKDs are activated and, in turn, induce autophosphorylation of tyrosine residues present in the C–terminus of the receptor tail (Figure 3). The phosphorylated tyrosines become docking sites for the SH2 (Src-homology 2) or PTB (phosphotyrosine-binding) domains of cytoplasmic signaling proteins, which initiate a signal transduction cascade resulting in the activation of transcription factors that upregulate the expression of genes involved in cell survival and proliferation [85]. 

The deregulation of kinase signaling pathways can be caused by different mechanisms. For example, the increased expression of RTKs on the cell membrane as well as mutations of the receptor or its downstream effector proteins can transduce mitogenic signals regardless of the ligand stimulation [86,87]. Much attention has been put on identifying potential agents against single components of RTK-related signaling pathways, in search of new effective tumor treatments [88]. Indeed, membrane receptor proteins account for around 70% of FDA-approved drug targets [89]. In particular, the accessible position of the receptor ectodomain, including the ligand-binding sites, has been considered an attractive target with which to block an aberrant RTK activation. Nowadays, there are two clinically approved cancer treatment strategies in place: monoclonal antibodies (mAb) and tyrosine kinase inhibitors (TKIs). Both approaches were designed to block the activities of the receptor and the enzymes of downstream signaling [90]. An alternative approach is the exploitation of receptor overexpression for the targeted administration of anticancer drugs. In this regard, the peptide ligands selected by phage display can be used to target an overexpressed RTK on tumor cells [91]. The peptide ligands of RTKs can directly bind the receptor extracellular domains and modulate their activities as agonist, antagonist, or allosteric modulators [41]. In addition, peptides can be conjugated to nanoparticles for tumor-targeted delivery of anticancer drugs [92]. For instance, different short and multicyclic peptides with high affinity binding for the epidermal growth factor receptor were discovered by phage display screening and represent a promising alternative for targeted drug delivery systems.

## 8. The Epidermal Growth Factor Receptor Family

The epidermal growth factor receptor (ErbB/HER) family represents the most studied type of RTK. It includes four structurally related members: Erb1 (EGFR), ErbB2 (Her2/Neu), ErbB3 (Her3), and ErbB4 (Her4) [93]. ERB1/EGFR was the first tyrosine kinase receptor to be discovered and linked to cancer [94]. It is a large transmembrane receptor (170–180 kDa) that contains four extracellular domains, two of which are cysteine-rich and responsible for ligand binding. EGFR can bind seven ligands, of which the epidermal growth factor (EGF) is the most studied [95]. The binding of the ligand to the receptor induces the homo- or hetero-dimerization with other members of the ErbB family, activating the cascade of intracellular events of receptor signaling. The main physiological role of EGFR is to regulate the development and homeostasis maintenance of the epithelial tissue [96]. Genetic alterations of EGFR are found in several tumors, including glioblastoma, lung, breast, gastric, and colorectal cancers. The aberrant activation of EGFR is caused by gene amplification, point mutations, in-frame deletions, or protein overexpression and contributes to the malignant transformation of epithelial cells [97]. Due to the main modulation of tumor cell growth, signaling, and survival [98], EGFR is a good candidate for tumor-targeted anticancer therapy [99]. Monoclonal antibodies, such as cetuximab and panitumumab, and tyrosine kinase inhibitors (TKIs) have been developed and approved for the treatment of EGFR-mutated non-small cell lung cancer (NSCLC), EGFR-expressing metastatic colorectal cancer (mCRC) with KRAS wild type, and head and neck cancers [100]. These therapies, alone or in combination, demonstrated an initial improvement in patient survival, but they were unfortunately followed by acquired drug-resistance due to new mutations and alternative bypass signaling pathways [101]. Various preclinical and clinical studies have also evaluated the effect of EGFR-TKIs in combination with other chemotherapeutic agents and therapies interfering with the proteins of downstream EGFR signaling in triple-negative breast cancer (TNBC) [102,103]. Great effort has been put to overcome the problems of acquired resistance to anti-EGFR therapies. In particular, several studies have been conducted for selecting peptide ligands of EGFR as promising tools for targeting overexpressed EGFR receptors in different cancer cell types [104]. For colorectal cancer treatment in the pre-, early-, and late-tumor phase, 17 EGFR-specific peptides were selected by phage display: QRHKPRE, HAHRSWS, YLTMPTP, TYPISFM, KLPGWSG, IQSPHFF, YSIPKSS, SHRNRPRNTQPS, NRHKPREKTFTD, TAVPLKRSSVTI, GHTANRQPWPND, LSLTRTRHRNTR, RHRDTQNHRPTN, ARHRPKLPYTHT, KRPRTRNKDERR, SPMPQLSTLLTR, and NHVHRMH (Patent WO 2016/029125 A1—Peptide reagents and methods for detection and targeting of dysplasia, early cancer, and cancer). In the photodynamic therapy of colorectal adenocarcinoma, the peptide QRHKPRE was conjugated with zinc phthalocyanine to be used for photodynamic therapy of colorectal adenocarcinoma [105]. The KLPGWSG peptide was used to target murine neural stem cells for nerve regeneration, as it stimulated cell differentiation toward the neuronal phenotype [106]. The IQSPHFF peptide was developed to detect NIR dyes that can be used for various applications, such as site-specific protein imaging and target molecule identification [107]. Moreover, this peptide had UV-responsive properties, inducing a morphological change by converting the peptide microparticles into nanotube forms [108]. By phage display screening using human hepatoma cells and human chronic myeloid leukemia cells, the phage clone encoding the peptide sequence YHWYGYTPQNVI (GE11) specific for EGFR was enriched [109]. The GE11 peptide conjugated with 18-fluorine was developed as a new radiolabeled peptide probe for imaging tumors that overexpress EGFR [110]. Two novel peptide ligands specific for EGFR were identified by phage display on an EGFR-overexpressing A-431 epidermal cell line. These peptides inhibited EGFR-induced phosphorylation in a concentration-dependent manner, and their binding was inhibited by the natural EGF ligand [111]. To provide a rapid method for producing antibodies for sensitive immunoassays, an immunoassay-like selection strategy was provided from a synthetic scFV phage display library to isolate antibodies against EGFR [112]. To identify monoclonal antibodies against EGFR currently used in cancer therapy, two peptides (P19 and P26), recognized specifically by panitumumab, were isolated by phage display. The recombinant fusion protein of P19/P26/heat shock-related protein (HSP) 70, used for immunization, induced the production of specific antibodies against the peptides and EGFR. This allowed consideration of these peptides as mimotopes suitable for vaccines for anti-EGFR immunotherapy in tumors [113]. HER2 is a 185-kDa transmembrane glycoprotein with an extracellular domain of 105 kDa (p105HER2 ECD). HER2 lacks a ligand, so it is activated upon homo- or hetero-dimerization with another ligand-occupied ErbB member or by proteolytic cleavage of its ECD [114]. HER2 expression is low in epithelial cells, while it is upregulated in cancer. HER2 protein overexpression and gene amplification represent a therapeutic target for breast and gastric/esophageal cancers. They are also found in the ovary, bladder, lung, head and neck, and colon cancers [115]. Different anti-HER2 therapies were developed: (i) humanized monoclonal antibodies, such as trastuzumab and pertuzumab, which inhibit the formation of receptor heterodimers by binding specific domains of the HER2 receptor; (ii) small TKIs, such as lapatinib, tucatinib, or neratinib, which block the intrinsic kinase activity; and (iii) antibody-drug conjugates (ADCs) that bind the receptor and mediate the entry of cytotoxic drugs into the tumor cells overexpressing HER2 [116]. Despite the therapeutic benefits in patients with HER2-positive breast or gastric cancers, the HER2 intra-tumoral heterogeneity and acquired drug-resistance were mainly responsible for treatment failure [117]. As an additional therapeutic strategy, phage display was used to identify HER2-specific peptide ligands to block the receptor activity [118]. Houimel et al. screened two random hexapeptide phage libraries and selected three linear peptides sequences (MARSGL, MARAKE, or MSRTMS), which specifically bound the ErbB-2 extracellular domain. To increase the peptide avidity, three anti-ErbB2 peptabodies were developed by fusion of the selected peptide with pentameric recombinant antibody; this peptide fusion was shown to inhibit in vitro cell growth of SK-BR-3 cells [119]. The peptide KCCYSL was also selected by phage display with high specificity for the ErbB-2 receptor, showing specific binding to breast and prostate cancer cells [120]. Another HER2-specific peptide (APTHER2) was conjugated with super-paramagnetic nanoparticles (SPION) and injected in HER2-positive tumor-bearing mice, in which it accumulated in the tumor mass, proving the in vivo binding ability to cancer cells [121]. In this regard, other Her2-targeted peptides have been analyzed in biomedical imaging and treatment as promising tools for imaging HER2-positive cancer patients [122]. 

## 9. Vascular Endothelial Growth Factor Receptor

The vascular endothelial growth factor receptor (VEGF) family and their ligands regulate the development, differentiation, and homeostasis of endothelial cells during normal and tumor-associated angiogenesis [123]. There are three types of receptors (VEGFR 1 to 3) that are expressed on the surface of normal endothelial cells in blood vessels and in hypoxic regions of tumor mass. The extracellular region contains seven immunoglobulin (Ig)-like domains, and only two of them, Ig-domains 2 and 3, are necessary for the binding to the specific ligand, causing the receptor dimerization [124]. The alternative splicing of VEGFR pre-mRNAs produces different protein isoforms with anti-angiogenic activities, which can be found as membrane-bound (mbVEGFR) or soluble (sVEGFR) forms [125,126]. The VEGF signaling has been implicated in the development of cancer and autoimmune diseases [127]. Indeed, the targeting of tumor vasculature emerged as a promising strategy for developing novel anti-angiogenic agents to block tumor growth and metastasis. Bevacizumab (avastin) was the first monoclonal antibody approved for the treatment of advanced colorectal cancer in combination with chemotherapy [128]. Other recombinant antibodies were selected by phage display to compete with the binding of VEGF to its receptor and counteract the VEGF-dependent tumor angiogenesis [129,130,131]. As potential alternative tools to monoclonal antibodies, a number of peptides have been selected by phage display for their ability to interfere with VEGF binding to VEGFR [132]. The screening of a heptapeptide library was performed with two different approaches in vitro using as bait a CHO ovary cell line expressing a recombinant KDR/VEGFR-2 or an anti-VEGF antibody. In vitro and in vivo assays demonstrated that the peptide ATWLPPR induced a complete inhibition of VEGF binding to cell-expressing KDR, inhibited the VEGF-dependent proliferation of endothelial cells, and completely repressed the VEGF-induced angiogenesis in a rabbit corneal model. Thus, the peptide ATWLPPR (also termed A7R) could represent a potential inhibitory agent for cancer-associated angiogenesis and metastasis [133]. The A7R peptide has been also analyzed for additional biomedical applications. In fact, it was radiolabeled for diagnosis based on imaging of tumor vasculature, or it was conjugated to nanocarriers for targeted drug delivery to normal and tumor endothelial cells [134]. In 2001, Giordano et al. developed an alternative selection method, known as BRASIL (biopanning and rapid analysis of selective interactive ligands), which was based on the sorting of cell-bounded phages from free phages by differential centrifugation. This additional separation step was used to screen a phage-displayed random peptide library with VEGF-stimulated HUVEC cells and to select the peptide CPQPRPL that bound to VEGFR1 and neuropilin-1 with higher specificity as compared to currently used selection methods. The motif PQPRPL was also demonstrated to be a chimeric VEGF-B-family mimic [135]. Another peptide K237-(HTMYYHHYQHHL) selected by phage display abrogated the VEGF/KDR interaction, inhibited the in vitro proliferation of primary endothelial cells, and showed anti-angiogenic properties in animal models in vivo [136]. Another selection of antagonizing peptides of VEGFR-1/Flt-1 receptor was performed by phage display dodecapeptide library using the recombinant receptor protein as bait. Among the seven selected peptides, F56 (WHSDMEWWYLLG) blocked VEGF binding to the Flt-1 receptor in vitro and inhibited blood vessel formation, tumor growth, and breast cancer metastasis in vivo [137]. As the pan-VEGF inhibitory peptides selected by phage display target all three members of VEGFR family, they may share common binding sites of these RTKs, and thus could be powerful anti-angiogenic drugs. 

## 10. Fibroblast Growth Factor Receptor

There are six subfamilies of secreted fibroblast growth factors (FGF-1, FGF-4, FGF-7, FGF-8, FGF-9, and FGF-19) that activate four FGF receptors (FGFR 1 to 4). The members of the FGFR family contain three extracellular Ig-like domains [138]. The binding of a ligand with a specific FGFR isoform activates the paracrine or endocrine signaling pathways that regulate various physiological and pathological processes, such as embryonic development, epithelial–mesenchymal interactions, metabolism regulation, migration, and angiogenesis [139]. FGFR is frequently mutated in genetic disorders, including Kallman’s syndrome [140], craniosynostosis syndromes [141], achondroplasia [142], and different cancers [143,144,145,146,147]. The selection of peptide ligands of hyper-expressed FGFR is certainly a strategic approach for cancer therapy. The LSPPRYP peptide was identified by phage display after three biopanning cycles using as bait murine fibroblasts overexpressing FGFR1c and FGFR2c isoforms and human keratinocytes overexpressing FGFR2. The LSPPRYP peptide competed with the natural ligand bFGF for binding to its receptor and showed strong anti-proliferative activity in vitro and in vivo. Therefore, this peptide was considered a promising candidate for anticancer therapy [148]. A phage-displayed library of 6-mer phage peptides was screened using Sf9 insect cells to select specific peptide ligands of FGFR1. The peptide VYMSPF was developed, which inhibited the mitogenic activity of aFGF, thus representing a good antagonist of aFGF for the therapy of human angiogenic diseases [149]. By screening a library of phage display heptapeptides on prostate cancer cells, Wang et al. identified the peptide HSQAAVP, named P12, which was a specific ligand of fibroblast growth factor 8b (FGF8b), antagonized the FGF8b activity and was useful for prostate cancer therapy [150]. The FGF peptide P7 (PLLQATL), showing high sequence homology to Ig-like domain IIIc of FGFR1 and FGFR2, was identified by screening a library of random heptapeptide by phage display using bFGF-stimulated murine fibroblasts [151]; this peptide inhibited cell proliferation by acting on the cell cycle and the Mitogen-Activated Protein Kinase (MAPK) signaling pathway [152]. The P7 peptide activity was investigated in different cancers, such as MDA-MB-231 breast cancer cells, in which proliferation was stimulated by bFGF and inhibited by P7 [153]. The therapeutic potential of P7 was also tested in bFGF-stimulated SKOV3 epithelial ovarian cancer cells, whose proliferation and migration were inhibited, suggesting that the P7 peptide could be a good candidate for targeted therapy of breast and ovarian cancers [154].

## 11. Platelet-Derived Growth Factor Receptor

The platelet-derived growth factor receptor (PDGFR) family includes four tyrosine kinase receptors (PDGF-A, -B, -C, and -D) that can homo- or hetero-dimerize [155]. The PDGFR consists of two subunits (alpha and beta), each one encoded by a different gene [156]. In the monomeric form, PDGFR is inactive; when PDGF binds to one of PDGFR isoforms (alpha or beta), the receptor dimerizes with three possible subunit combinations, named -αα, -ββ, and -αβ. The extracellular region of the receptor consists of five immunoglobulin-like domains, and the intracellular region contains a tyrosine kinase domain. PDGF-BB is the only PDGF ligand that can bind all three combinations of receptors with high affinity [157]. The cellular expression of PDGFR isoforms is variable as some cells display only one of the PDGFR isoforms while others express more isoforms. PDGF/PDGFR isoforms regulate several cell proliferation processes, differentiation, and apoptosis [158]. They promote the differentiation of mesenchymal cells, such as fibroblasts and pericytes, the main components of the tumor microenvironment involved in promoting tumor proliferation, metastasis, and response to therapy [159]. PDGFRβ is highly expressed in many tumors, playing a key role in activating angiogenesis and regulating tumor interstitial fluid pressure [160,161,162]. The decreased interstitial fluid pressure with consequent improved trans-capillary molecular transport was the basis for new therapeutic approaches [163]. The role of PDGFRβ in various pathophysiological mechanisms makes this receptor a promising candidate for targeted therapy of tumors associated with high expression of PDGF-BB [164]. Although many PDGFRβ inhibitors have been developed and tested, most of these inhibitors were not specific for PDGFRβ and showed activity to other kinases [165]. The monoclonal antibody 2AIE2 has been developed against the extracellular region of the PDGFR. It is particularly mapped to the fifth Ig- domain of the PDGF-beta receptor, which implies that this domain is essential for inhibiting the binding between ligand and receptor [166]. In addition to monoclonal antibodies for therapeutic targeting, a new approach uses peptides with specific targeting characteristics. To identify human PDGFRβ-specific binding peptides, Askoxylakis et al. performed biopanning using as bait the immobilized recombinant extracellular domain of PDGFRβ and subsequently the biotinylated form in suspension. After four rounds of selection, 40% of the clones isolated on the immobilized protein and 80% of the clones isolated on the biotinylated target in suspension showed the peptide sequence IPLPPPSRPFFK [167]. To determine the properties of PDGFR-P1 on tumor cells, the peptide binding was evaluated on human pancreatic and breast cancer cells that overexpress PDGFRβ. The in vivo biodistribution studies showed a more significant accumulation of the peptide in the tumor than in most healthy tissues. This study suggested that the peptide PDGFR-P1 could be useful for developing novel peptide-based ligands of platelet-derived growth factor receptor beta for tumor monitoring. There is still very little information about the role of the PDGF-B/PDGFR-β axis in aggressive triple-negative breast cancer (TNBC) subtypes [168]. Applying phage display to select peptide ligands of PDGFR expressed in TNBC could be useful for developing original reagents for interfering with the receptor signaling, and for getting new insights into the molecular mechanisms of this cancer type.

## 12. Discussion

It is well known that tyrosine kinase receptors play crucial roles in cell growth and differentiation and in cancer, as they act as important mediators for a myriad of cell signaling pathways. For this reason, RTKs represent a prominent class of receptors as therapeutic targets in a variety of pathological diseases. Remarkably, understanding mechanisms that cause abnormal activation of RTKs in cancers attracted researchers to study new techniques for discovering innovative drugs. Several monoclonal antibodies target the extracellular domain of these receptors, and small tyrosine kinase inhibitors, which target the ATPase intracellular kinase domain or downstream effector proteins, are under pre-clinical and clinical studies or already FDA-approved. However, there are still critical points to address, such as drug resistance, target selection, and immunogenicity. The search for peptide ligands of RTKs was undertaken to overcome these problems as a new strategy for targeting overexpressed RTKs in cancer cells [169]. The use of peptides was first associated with many drawbacks: the hydrophilic nature of peptides and their conformational flexibility, which sometimes led to problems in crossing some physiological barriers; the necessity of administration by injection because oral administration and the presence of proteolytic enzymes in the digestive tract causes a rapid degradation; and peptides’ quick hepatic and renal elimination from the blood circulation [170]. Despite the problems of in vivo stability and short half-life, the peptides have multiple advantages for drug discovery and therapeutic applications. In fact, they are characterized by small size, bioactivities, and safety from adverse reactions of the immune system. A growing number of studies have been conducted to improve the chemical properties of peptides and their targeted delivery toward a specific in vivo target [171]. Innovation in the study of peptides was the introduction of phage display technology, which in 2018 won the Nobel Prize in Chemistry, allowing the selection of specific ligands from phage libraries that expose thousands of random peptides on their surface coat. There are various methods of biopanning screening and selection of peptide binders with high affinity [172]. Thanks to this technique it has been possible to identify specific ligands of membrane receptors, and therefore to study the modulation of related signaling pathways [173]. This allowed the discovery of peptide candidates for targeted tumor therapies and, in some cases, early monitoring of cancer. In this review, we examined novel peptides specific to the main RTKs families ErbB, VEGFR, FGFR, and PDGFR. All these peptides have been identified by phage display libraries screening and have shown potential application in oncological therapy [40]. As these RTKs are overexpressed in different cancer types, showing aggressiveness and decreased patient survival, the peptides that negatively modulate the activity of these receptors are useful models for drug development [174]. Further, the conjugation of peptide ligands with nanocarriers can represent a new potential drug delivery system in cancer therapy.

## Figures and Tables

**Table 1:** Specific characteristics of the main bacteriophages used in phage display.

Bacteriophage	Length	Size	Genome	Proteins		Displayed Copies/ Virions		Viral Life Cycle
M13	930 nm	6.4 kb	ss DNA	Replication proteins	pII, pX, pV	3–5 on pIII	110 Kda	Lysogenic
				Morphogenetic proteins	pI, pIV	2700 on pVIII	10 Kda	
				Structural proteins	pIII, pVIII, pVI, pVII, pIX			
T7	Head 55 nm	40 kb	ds DNA	Capsid proteins	gp10A, gp10B	Up to 1200 polypeptide	132 Kda	Lytic
				Inner core proteins	gp16, gp15, gp14			
	Tail 19 nm			Connector proteins	gp8, gp6, gp7, gp11, gp12	1–415 peptide		
				Tail protein	gp17			
T4	90 nm wide	168 kb	ds DNA	Head protein	gp20, gp23, gp24	810 copies of polypeptides on SOC proteins	710 Kda	Lytic
	200 nm long			Tail protein	gp15, gp13, gp14, gp18, gp19, gp34, gp35, gp36, gp9, gp10, gp11, gp12	155 copies of polypeptides on HOC proteins		
Lambda	Head 64 nm	48.5 kb	ds DNA	Head protein	gpD, gpE, gpC, gpB, gpW	405 on pD proteins		Lysogenic/ lytic
	Tail 150 nm			Tail protein	gpU, gpV, gpJ, gpH	6 copies on pV proteins		
